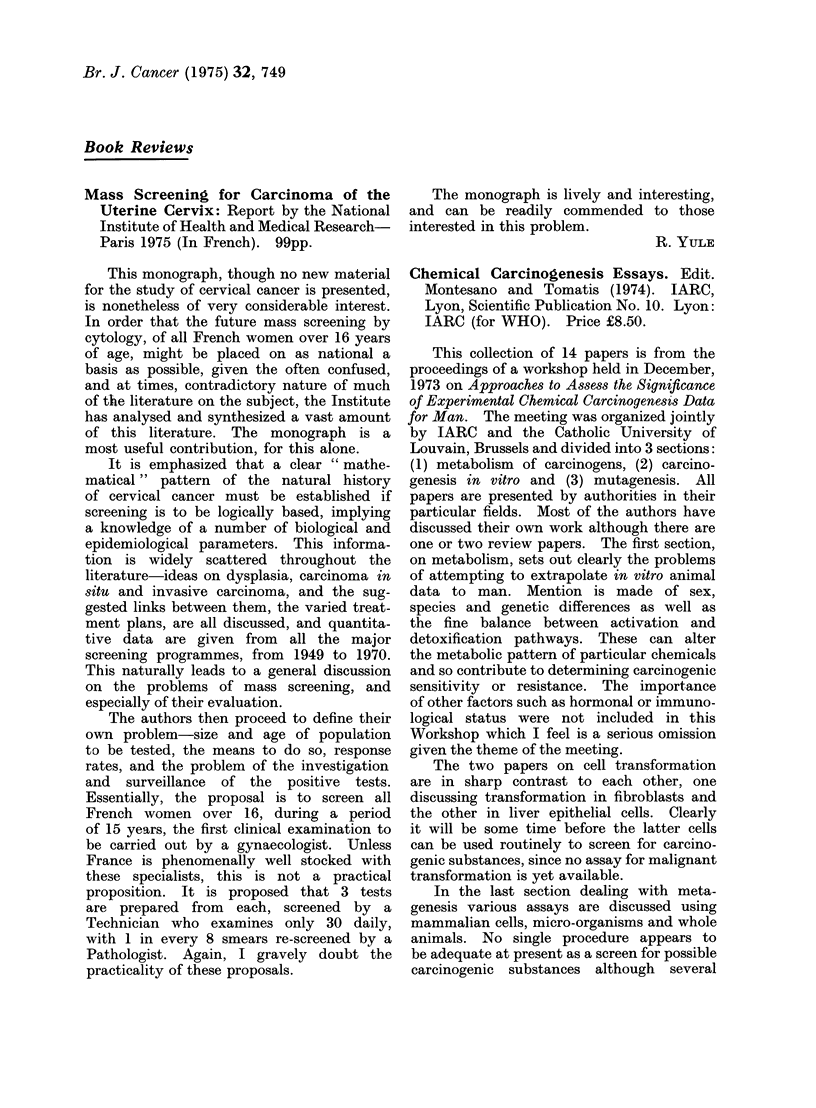# Mass Screening for Carcinoma of the Uterine Cervix

**Published:** 1975-12

**Authors:** R. Yule


*Br. J. Cancer* (1975) **32**, 749

### *Book Reviews*

**Mass Screening for Carcinoma of the Uterine Cervix:** Report by the National Institute of Health and Medical Research—Paris 1975 (In French). 99pp.

This monograph, though no new material for the study of cervical cancer is presented, is nonetheless of very considerable interest. In order that the future mass screening by cytology, of all French women over 16 years of age, might be placed on as national a basis as possible, given the often confused, and at times, contradictory nature of much of the literature on the subject, the Institute has analysed and synthesized a vast amount of this literature. The monograph is a most useful contribution, for this alone.

It is emphasized that a clear " mathematical " pattern of the natural history of cervical cancer must be established if screening is to be logically based, implying a knowledge of a number of biological and epidemiological parameters. This information is widely scattered throughout the literature—ideas on dysplasia, carcinoma *in situ* and invasive carcinoma, and the suggested links between them, the varied treatment plans, are all discussed, and quantitative data are given from all the major screening programmes, from 1949 to 1970. This naturally leads to a general discussion on the problems of mass screening, and especially of their evaluation.

The authors then proceed to define their own problem—size and age of population to be tested, the means to do so, response rates, and the problem of the investigation and surveillance of the positive tests. Essentially, the proposal is to screen all French women over 16, during a period of 15 years, the first clinical examination to be carried out by a gynaecologist. Unless France is phenomenally well stocked with these specialists, this is not a practical proposition. It is proposed that 3 tests are prepared from each, screened by a Technician who examines only 30 daily, with 1 in every 8 smears re-screened by a Pathologist. Again, I gravely doubt the practicality of these proposals.

The monograph is lively and interesting, and can be readily commended to those interested in this problem.

R. YULE